# Coadjuvants in the Diabetic Complications: Nutraceuticals and Drugs with Pleiotropic Effects

**DOI:** 10.3390/ijms17081273

**Published:** 2016-08-05

**Authors:** Thiago Melo Costa Pereira, Fabio Silva Pimenta, Marcella Lima Porto, Marcelo Perim Baldo, Bianca Prandi Campagnaro, Agata Lages Gava, Silvana Santos Meyrelles, Elisardo Corral Vasquez

**Affiliations:** 1 Pharmaceutical Sciences Graduate Program, Vila Velha University (UVV), Av. Comissario Jose Dantas Melo 21, Boa Vista, 29102-920 Vila Velha, Brazil; pereiratmc@gmail.com (T.M.C.P.); drfabiospimenta@hotmail.com (F.S.P.); biancacampagnaro@yahoo.com.br (B.P.C.); 2 Federal Institute of Education, Science and Technology (IFES), 29106-010 Vila Velha, Brazil; cella.porto@gmail.com; 3 Burn Treatment Center, Children State Hospital, 29056-030 Vitoria, Brazil; 4 Department of Pathophysiology, Montes Claros State University, 39401-089, Montes Claros, Brazil; marcelobaldo@ymail.com; 5 Laboratory of Translational Physiology, Federal University of Espirito Santo (Ufes), 29047-100 Vitoria, Brazil; agatagava@hotmail.com (A.L.G.); meyrelle.vix@terra.com.br (S.S.M.); 6 Division of Nephrology, McMaster University, Hamilton, ON L8N 4A6, Canada

**Keywords:** diabetes, quercetin, polyphenols, resveratrol, silymarin, kefir, probiotic, sildenafil, phosphodiesterase inhibitors, antioxidants

## Abstract

Because diabetes mellitus (DM) is a multifactorial metabolic disease, its prevention and treatment has been a constant challenge for basic and clinical investigators focused on translating their discoveries into clinical treatment of this complex disorder. In this review, we highlight recent experimental and clinical evidences of potential coadjuvants in the management of DM, such as polyphenols (quercetin, resveratrol and silymarin), cultured probiotic microorganisms and drugs acting through direct/indirect or pleiotropic effects on glycemic control in DM. Among several options, we highlight new promising therapeutic coadjuvants, including chemical scavengers, the probiotic kefir and the phosphodiesterase 5 inhibitors, which besides the reduction of hyperglycemia and ameliorate insulin resistance, they reduce oxidative stress and improve endothelial dysfunction in the systemic vascular circulation. In the near future, experimental studies are expected to clear the intracellular pathways involving coadjuvants. The design of clinical trials may also contribute to new strategies with coadjuvants against the harmful effects of diabetic complications.

## 1. Introduction

Diabetes mellitus (DM) is an important public health issue because it is highly associated with increased morbidity and mortality [1]. In fact, the prevalence of diagnosed diabetes is increasing worldwide, as demonstrated by the rise from 6.5% (1999 to 2002) to 7.8% (2003 to 2006) of the population in just a few years [2]. Type 2 DM is the most common form of the disease and affects 90% to 95% of individuals with diabetes. The main issue of this pandemic is the increase in mortality associated with diabetes due to the risk of cardiovascular diseases (CVD), which are the leading cause of death in this population. This information became clear after an analysis of the First National Health and Nutrition Examination Survey (NHAHES), which covered the period of 1971–1993 and revealed that more than 65% of deaths of people with diabetes were due to CVD [3]. In addition, diabetes is a leading cause of morbidity and leads to microvascular and macrovascular complications [3,4,5].

Even with the already reported increase in the prevalence of diabetes over the years, only approximately 13% of individuals diagnosed as diabetic were in conformity with the control of the established levels of serum glucose, blood arterial pressure and total cholesterol at the same time [6]. Indeed, it is well known that most of the type 2 DM fail to control glycemia to normal levels when subjected only to diet and physical exercise and, consequently, it is necessary to treat them with anti-diabetic pharmacotherapy [1]. For instance, in a period of two years, among those patients who have been diagnosed as diabetics, the percentages of success in the control of glycated hemoglobin, blood pressure and total cholesterol are higher than 7%, 35% and 37%, respectively. As a result, good management of type 2 diabetes with pharmacological as well as non-pharmacological therapy (including reduction of caloric intake and intermittent fasting) is important [7,8]. Lately, with the mission of ameliorating this health problem, eight different classes of drugs for treatment of type 2 DM, with variations in their side effects and costs, have been approved by the US Food and Drug Administration (FDA) [9]. In this regard, investigators have been challenged to test potential therapies for DM based on functional foods, which are of low cost and very accessible (e.g., substances derived from marine algae [10]).

In the present review, we discuss some epidemiological aspects of diabetic complications resulting from hyperglycemia and the therapeutic advances with antioxidant substances based on experimental and clinical studies. Among different alternatives discussed in this review, we highlight the putative coadjuvants in the management of DM, such as functional foods rich in polyphenols and the probiotic kefir. In addition, we discuss drugs with pleiotropic effects, such as phosphodiesterase 5 (PDE5) inhibitors, which lately have been the main focus of investigation in our laboratory.

## 2. The Impact of Chronic Hyperglycemia on Diabetic Complication

### 2.1. Epidemiological Aspects of Diabetes

The high mortality and morbidity observed in DM patients characterized by chronic high levels of blood glucose and HbA1c, which compromises the function of the target organs heart and kidneys [11,12]. Therefore, the desired goal of treatment for diabetes is to maintain euglycemic levels as much as possible. Studies from early last century have also highlighted several effects of uncontrolled diabetes, such as dyslipidemia [13], reduced serum protein [14], skeletal muscle changes [15], and other complications. In the last decade, several epidemiological studies have been conducted to identify the risks associated to diabetes.

It is well known that diabetes doubles the risk for acute coronary syndrome with an additional risk once the event has occurred. This risk was evident in the Tehran Lipid and Glucose Study, a population-based cohort study that took place in Iran. The authors found that in type 2 diabetic patients, hypercholesterolemia and central adiposity were independent risk factors for death by cardiovascular causes, and poor glycemic control is an independent risk factor for both cardiovascular and all-cause mortality [16]. In the United Kingdom, a cohort study of myocardial infarction risk in men and women with and without diabetes was carried out using a large, nationwide primary care database. The overall adjusted relative risk of myocardial infarction was higher in individuals with diabetes versus no diabetes and was greater in women compared to men [17].

After a 23-year follow-up to determine the prevalence of diabetes and associated characteristics, the Da Qing IGT and Diabetes Study showed that CVD was the leading cause of death in individuals with diabetes (47.5% in men and 49.7% in women), and almost half of the deaths were due to stroke [18]. This excessive risk of stroke associated with diabetes was significantly higher in women than men, and there were no sex differences for other major cardiovascular risk factors [19]. It is noteworthy that high glucose levels alone did not account for the increased risk associated with diabetes. A meta-analysis of 15 prospective studies in approximately 760,000 patients showed that people with pre-diabetes, which was defined as impaired fasting glucose of 110 to 124 mg/dL (6.1 to 6.9 mmol/L) or both, exhibited a moderate higher risk of stroke events [20].

Another condition associated with diabetes is chronic kidney disease. In fact, diabetes is one of the leading causes of chronic kidney disease in the United States [21], where the prevalence of diabetic nephropathy in the population of patients with type 1 and 2 DM is 20% to 40% [22,23]. The Madrid Diabetes Study, which is a prospective cohort study of 3443 type-2 diabetic outpatients, showed that the unadjusted hazard ratio for all-cause mortality in diabetic patients with eGFR < 60 mL/min/1.73 m^2^ was approximately 3 after five years of follow-up. Patients with chronic kidney disease at baseline had an increased risk of cardiovascular mortality [24].

### 2.2. Toxic Effects of Hyperglycemia

Chronic hyperglycemia can promote toxic effects in a myriad of tissues, especially in neurons, because they are more susceptible to glucose uptake [25].

Uncontrolled diabetes cam result in a pathological state characterized by severe hyperglycemia, elevation of plasma osmolarity and diabetic ketoacidosis [26]. Its classic manifestation consists of the biochemical triad of hyperglycemia, increased ketones in bloodstream, and metabolic acidosis, and it might be caused by several factors, including reduced secretion and action of insulin, and raised levels of anti-insulin hormones [27,28].

In general, patients with chronic hyperglycemia exhibit many other characteristics, such as altered expression of matrix degrading enzymes, increased synthesis and deposition of extracellular matrix (ECM), generation of advanced glycation end products (AGE), upregulation of pro-inflammatory cytokines and growth factors, and augmented flux of hexosamines and polyols [29]. Moreover, in chronic hyperglycemia conditions, the augmented glycation of intracellular proteins appears to attack other proteins and worsen the exacerbate formation of AGEs [30,31]. Consequently, it leads to the inhibition of mitochondrial respiration, increased production of reactive oxygen species (ROS) and inflammatory cytokines, culminating with marked alterations in the systemic vascular function. Also, it is well known that the augmented production of ROS causes DNA damage and results in alteration in the expression of ECM glycoproteins, which corroborates the concept that augmented oxidative stress accounts for DM complications [32,33].

In chronic hyperglycemia, aldose reductase is activated and catalyzes the first reaction in the polyol pathway, resulting in exacerbated productions and accumulation of sorbitol [34,35,36] and causing cellular toxicity by osmotic effects. NADPH is consumed and NADH is produced with accumulation of sorbitol and fructose that can also affect cellular osmosis. While there is an oversupply of NADH in individuals with diabetes due to chronic hyperglycemia and enhanced fatty acid oxidation, NAD^+^ could be depleted due to the activation of poly ADP ribose polymerase (PARP) by oxidative DNA damage during oxidative stress [37,38].

The activation of apoptosis in chronic hyperglycemia has received much attention in recent years. Several mechanisms regulate the complicated signaling pathways that mediate apoptosis by hyperglycemia. This process is initiated by interruption of mitochondrial electron transport, resulting in an incomplete reduction of molecular oxygen, generating superoxide anion (·$O_{2}^{-}).$ This free radical can react with nitric oxide (NO), resulting in the production of peroxynitrite (ONOO^−^), which is a highly toxic molecule [39,40] that causes endothelial cell death. Dysfunction of endothelial cells, which causes loss of multiple endothelium-derived substances, has been hypothesized to play a key role in the progression of vascular disease in diabetes [41,42].

### 2.3. Role of Oxidative Stress in Diabetic Complications

Oxidative stress is induced by elevations in glucose and free fatty acid levels and has a key role in the pathogenesis of both types of DM and on diabetic complications, as has been reviewed by Wei et al. [43]. Recent evidence suggests oxidative stress is a key participant in the development and progression of diabetes as well as its micro- and macrovascular complications [44,45,46]. Paradoxically, not much attention has been given to other possible therapeutic interventions besides glucose reduction.

ROS are a group of short-lived molecules derived from aerobic respiration and other oxygen reactions that include ·$O_{2}^{-}$, hydrogen peroxide (H_2_O_2_), hydroxyl radical (·OH), ONOO^−^ and hydroxyl (OH^−^) [47,48]. The major sources of ROS are the mitochondria, NADPH oxidases, xanthine oxidase, uncoupled NO synthase (NOS), lipoxygenase, cyclooxygenases and CYP450, but they vary in their pathological role and importance depending on the disease and the organ [40,49]. Mitochondria and NADPH oxidases (Nox) are the most important sites for ROS production and are responsible for cardiovascular complications in diabetes [50]. In 1999, Ide et al. [51] observed enhanced cardiomyocyte mitochondrial ·$O_{2}^{-}$ in the failing myocardium. Moreover, Selemidis et al. [52] suggested that NADPH is a primary ROS-producer not only in vascular smooth muscle cells but also in cardiomyocytes, vascular endothelial cells and adventitial fibroblasts. Furthermore, increased expression of Nox isoforms has been associated with myocardial hypertrophy and fibrosis in diabetes [52,53].

Hyperglycemia is characterized not only by a high-level production of ROS but also by an impairment of the intracellular antioxidant defense system, such as the nuclear factor (erythroid-derived 2)-like 2 (Nrf2), a master upregulator of several antioxidant enzymes [54,55]; consequently, the induction of genes encoding antioxidant molecules, including superoxide dismutase (SOD), glutathione peroxidase (GPx) and catalase is also affected [56]. Additionally, reduced SOD, catalase and GPx activity have been reported in both experimental and clinical diabetic conditions due to excessive glycation [57,58]. Batinic-Haberle et al. [59] found that diabetic blood vessels exhibited an improved endothelium-dependent relaxant response when treated with SOD. Interestingly, a recent study showed that the antioxidant curcumin may have a protective role against oxidative stress in diabetic mice (*db*/*db*) [60]. Therefore, it is important to emphasize that the nutraceutical compounds that require the activation of Nrf2 have been considered as relevant therapeutic strategy for prevention/treatment of diabetic complications [55,56].

Regardless of the imbalance between the generation of ROS and the activity/intracellular levels of the antioxidant defense mechanisms, excessive generation of ROS is a deleterious factor that leads to pathological consequences, including irreversible cellular damage by oxidation of proteins, lipids, carbohydrates and nucleic acids [61]. Recent evidence indicated that increased levels of urinary markers of oxidative DNA and RNA damage occur with diabetic complications [62]. Furthermore, Palem and Abraham [63] observed that diabetic patients taking both oral antidiabetic drugs and insulin still present high levels of oxidative stress, which emphasizes the need for adding antioxidants to reduce the impact of diabetic complications.

In addition to the direct damage to cells, increased ROS levels also cyclically activates pathways associated with diabetes complications, such as the polyol pathway, increased production of AGEs, activation of PKC isoforms and the hexosamine pathway [54,64]. ROS overproduction and increased oxidative stress can also cause vascular endothelial and smooth muscle dysfunction. On the other hand, it has been shown that neutralization of reactive molecules in patients with diabetes was capable of preventing cardiomyopathy, retinopathy, nephropathy and neuropathy [65]. To avoid diabetes disorders, hyperglycemia should be treated promptly through stimulation of insulin secretion (not the best choice) or increasing insulin sensitivity. However, adopting a causal antioxidant therapeutic approach might be a modern adjuvant strategy to prevent the overproduction of ROS and consequently complications from diabetes.

## 3. Potential of Natural Products with Antioxidant Effects for Treating Diabetes

Polyphenolic compounds are widely found in plants and provide several pharmacological properties, including antidiabetic effects [66,67,68]. Although not focused in the present discussion, it is important to recognize that the Chinese medicine has demonstrated the efficacy of several natural products that have been used in the treatment of DM as reviewed elsewhere [69]. In this subsection, the main polyphenols with potential antidiabetic activity investigated by us as well as others will be addressed.

### 3.1. Polyphenolic Compounds

#### 3.1.1. Quercetin

Quercetin (2-(3,4-dihydroxyphenyl)-3,5,7-trihydroxychromen-4-one) is the major flavonoid involved in vegetables and fruits, and it exhibits metabolic, anti-oxidative, anti-apoptotic and renoprotective effects at adequate doses [45,46,68,70]. Although this molecule is widely consumed in the diet, it was surprisingly reported as mutagenic in the 1970s in a study with unusual methods and with no reproducible results [70]. Only in 1999 did the International Agency for Research on Cancer conclude that quercetin should not be classified as carcinogenic to humans [70]. However, in parallel, the investigations with quercetin related to diabetes began in 1975 with an initial interest in preventing cataracts through the inhibition of the aldose reductase that blocks polyol accumulation in intact lenses [71]. Only in the 1990s did quercetin studies extend to other targets in diabetes complications.

Many studies have demonstrated that this bioflavonoid may act through diverse pathways to decrease the tissue-damaging effects of chronic hyperglycemia, such as stimulation of glucose uptake via GLUT4 [72,73,74], inhibiting hepatic glycogenolysis and gluconeogenesis [72,75], and inhibiting α-glucosidase in the small intestine [76] or intestinal glucose transporter GLUT2 [77]. At the same time, another potential advantage is that quercetin exhibits all the characteristics of an adequate antioxidant for diabetes treatment: free radical scavenger ability [78,79], long half-life (~20 h in humans) [80,81], capacity to suppress pro-oxidant enzymes (NADPH oxidase, xanthine oxidase and CYP) [82,83,84] and the ability to stimulate antioxidant enzymes (SOD, catalase, glutathione peroxidase and glutathione reductase) [68,85,86] with high mitochondrial permeability [46,87], which are an important source of ROS in diabetes [88,89]. Given these multiple potential mechanisms, quercetin becomes an important protective molecule against the consequences of long-term diabetes (e.g., microvascular and macrovascular damage, nephropathy, neuropathy associated with the risks autonomic disturbance, amputations and foot ulcers) [67,90,91], as demonstrated in the experimental and clinical investigations as discussed below.

In streptozotocin (STZ)-induced type 1 diabetes models, varying doses of quercetin have shown several benefits. At 50 mg/kg/day (oral dose), quercetin prevented retinal degeneration [92] and vascular complications by inhibiting NF-κB signaling [93]. In rats, quercetin ameliorated erectile dysfunction by inhibiting oxidative stress and upregulating eNOS [94], and it protected against the progression of neuropathy even with a low dose of quercetin (10 mg/kg) as well as attenuating cold allodynia and hyperalgesia [95]. Recently, for the first time, we demonstrated that the same low dose of quercetin attenuates hyperglycemia and nephropathy in STZ-induced diabetes in apolipoprotein E-deficient mice [46] and in C57BL/6J mice [45] (or in rats in a study conducted by others [96,97]), and quercetin treatment exhibited antioxidant benefits. With different doses (25 to 100 mg/kg/day), quercetin was also capable of suppressing the kidney inflammatory response at least partly via anti-hyperuricemic and anti-dyslipidemic effects [98]. In *db*/*db* mice (the most popular mouse model for type 2 DM), quercetin also demonstrated satisfactory effects [76]. At doses ranging between 50 and 100 mg/kg/day, quercetin treatment improved postprandial blood glucose (similarly to acarbose) [76] in addition to avoiding hyperglycemia and hyperlipidemia and increasing the antioxidant status [99]. Although experimental studies clearly support the protective effects of quercetin in diabetes, clinical data with this isolated compound are still insufficient and inconclusive. Recently, 500 mg of daily quercetin (for four weeks) was capable of reducing hyperuricemia in healthy men [100], which is a relevant factor associated with insulin resistance and progression of diabetic complications [91]. On the other hand, quercetin administered at the same dosage in women with type 2 DM, has been shown to decrease systolic arterial pressure, without significant effects on other cardiovascular risk factors [101]. Similarly, recent data from Brüll et al. [102] revealed that quercetin (162 mg/day) decreased day- and nighttime systolic blood pressure in overweight-to-obese patients without changing any other metabolic risk factor. More recently, another study reported no effect on flow-mediated dilation or insulin resistance with an analogue of quercetin (quercetin-3-glucoside, at 160 mg/day) in healthy men and women aged 40–80 years [103]. Therefore, more studies about quercetin will be necessary to establish the ideal dosage and to identify the real efficacy in diabetic patients.

#### 3.1.2. Resveratrol

This non-flavonoid polyphenolic compound (3,5,4′-trihydroxystilbene, notably present in peanuts, grapes, grape juice and red wine) might be the main molecule responsible for cardiovascular protective effects in the French population despite a high intake of saturated fats, which is known as “French Paradox” [66,104,105,106]. For that reason, this potent molecule (even with a short half-life) also would be highly beneficial as an adjuvant therapy for diabetes. Additionally, under in vitro [107,108] and in vivo [109,110,111] experimental conditions that mimic human diabetes, resveratrol has been shown to have a potential benefit in several multi-target mechanisms for diabetic complications, as presented below.

Recently, Yan et al. [112] showed that 40 mg/kg/day of oral resveratrol (a high dose—according to Zhou et al. [112]) reduced proteinuria and attenuated the progress of renal fibrosis in *db*/*db* mice [112,113]. At the other extreme, it was demonstrated that a low dose of oral resveratrol (0.5 mg/kg) ameliorated classical DM symptoms (e.g., polydipsia, polyphagia, and body weight loss) and delayed the onset of insulin resistance in an STZ model [66], which probably occurred through improved glucose homeostasis. This evidence was supported by Palsamy et al., who in 2009 [114] showed decreased activity of key enzymes for gluconeogenesis by treating rats with mild diabetes (STZ-nicotinamide model) with a low dose (5 mg/kg) of resveratrol. Moreover, several in vitro studies have shown that resveratrol can increase glucose uptake by targeting insulin-affected cells (skeletal muscle, adipocytes and hepatocytes) [115,116,117,118,119], thereby improving the insulin signaling probably through improvement of insulin sensitivity in a SIRT1-dependent manner [120,121,122] or by other distinct mechanisms [66]. This stimulation of SIRT1 (a pivotal mediator of the metabolic effects of resveratrol) also may promote an increase of antioxidant enzymes (SOD, catalase, GPx and glutathione-*S*-transferase) in pancreatic β-cells and decrease the function of pro-inflammatory mediators (IL-6, NF-kB and COX-2) in many diabetic target tissues [119,123,124,125], which explains the relevant protective effects against apoptosis, neurodegeneration and cardiovascular complications [106,126,127]. Interestingly, resveratrol also seems to contribute to endothelial repair (which is an important tissue affected by chronic diabetes) through free-radical scavenging and/or restoration of eNOS functionality that culminates with increased bioavailability of NO [106,109,127,128] and consequently reduces diabetic complications. Recently, Neves et al. [129] showed another possible pathway of cellular protection through the regulation of cell membrane structure and fluidity (similar to cholesterol). In addition, it was observed that resveratrol might reduce endoplasmic reticulum stress by avoiding misglycosylation, depletion of calcium stores and DNA damage [127]. Therefore, resveratrol not only acts by glycemic control per se but also provides antioxidant and other pleiotropic effects [125,129,130].

Even though the preclinical evidence includes experimental evidence that clearly demonstrated that resveratrol has a significant antidiabetic effect in a wide dose range (0.1 to 1.500 mg/kg body weight), recommending resveratrol as a therapeutic supplement or treatment for diabetes patients is still controversial, and there is a similar controversy for quercetin [119,127,131,132]. This is a problem because it has generated serious doubts about the potential usefulness of these substances, particularly for dietary prevention strategies [133,134,135,136]. For instance, Thazhath et al. [137] have recently demonstrated that 1000 mg/day resveratrol in diet-controlled type-2 DM patients for five weeks did not change body weight, glycemic control or GLP-1 secretion. Similar data were also obtained by Poulsen et al. [132], who gave 1500 mg/day of resveratrol for four weeks to obese patients and found no effects on metabolic biomarkers, blood pressure or resting energy expenditure. These apparent unsuccessful studies also may be explained by variability between volunteers (age, body weight, nutrition, severity of diabetes) and/or duration of treatments [119]. In agreement with this hypothesis, another study of patients with metabolic syndrome treated the patients with 1500 mg/day of resveratrol for 90 days (~13 weeks) and revealed a significant reduction in body weight and insulin secretion [137]. Additionally, Goh et al. [138] showed improvement of insulin sensitivity via SIRT1 through 3000 mg of resveratrol for 12 weeks in type-2 diabetic patients. The advantage of this regulation is the promotion of survival and longevity, associated with telomere length [7]. Even for a shorter period of time, it was shown that resveratrol (1000 mg daily in first week followed by 2000 mg daily in second week) was able to reduce hepatic and intestinal lipoprotein production [139]. It is important to consider that although there are several investigations on the tolerability of resveratrol in humans, we cannot ignore the fact that studies about long-term resveratrol toxicity (or analogues such as pterostilbene) are still needed.

#### 3.1.3. Silymarin

Silymarin is a dry flavonoid mixture extracted after processing the seeds of *Silybum marium* with ethanol, methanol, and acetone [140], which contains seven major components: taxifolin (the most effective antioxidant), silychristin, silydianin, silybin A, silybin B, isosilybin A and isosilybin B [141,142]. Although silymarin has mainly been used to treat liver diseases [143], its antidiabetic activity was recently reported and is associated with an anti-glycation profile [144,145], inhibition of aldose reductase [143], partial agonist activity in peroxisome proliferator-activated receptor γ (PPARγ) [143], antioxidant capacity and radical scavenging [144,146]. All these characteristics make silymarin an interesting candidate for the prevention and treatment of diabetic complications, which has recently been demonstrated both in experimental models and in humans (the same as for quercetin and resveratrol).

In 2013, Sheela et al. [146] demonstrated more fully that silymarin (60 and 120 mg/kg/day, i.m., for eight weeks) was able of reduce the classical signs of DM and attenuate the progression of the disease in a STZ-nicotinamide-induced nephropathy model (although the possible pathways were not investigated). However, in parallel, an in vitro study revealed that podocytes exposed to high glucose restored the ·$O_{2}^{-}$ production and NADPH oxidase activity to basal levels through 10 μM of isolated compound silybin. In the same paper, Khazim et al. [147] obtained similar results in an in vivo experiment using 100 mg/kg/day of same substance (i.p., six weeks) in an advanced new model of diabetic nephropathy (OVE26 mice) with an additional reduction in albuminuria. These data also corroborated the findings of Vessal et al. [148], who used silymarin (100 mg/kg/day, i.p., for four weeks) in an STZ-rat model to obtain a reduction in kidney lipid peroxidation and increase the activity of catalase and GPx under hyperglycemia conditions.

Further studies are necessary to explore the other cytoprotective effects of silymarin. For example, Tuorkey et al. [140] recently showed that this flavonoid mixture (120 mg/kg, i.p., for 10 days) could protect cardiomyocytes against apoptosis in diabetic (alloxan) rats via restoration of caspase-3 and Bcl-2 to control levels. For neuroprotection, silymarin (100 mg/kg/day for eight weeks) ameliorated hyperalgesia and sciatic motor nerve conduction velocity in STZ-diabetic neuropathic rat by reducing lipoperoxidation and increasing SOD activity [149]. Moreover, silibinin in *db*/*db* mice provided DNA protection and reduced oxidative stress in a brain-specific area in rodents [150]. Therefore, these preliminary studies also revealed the potential of silymarin as a valid tool to counteract oxidative stress in the central nervous system under diabetic conditions [151].

Although there are still only a few clinical studies with silymarin, the results have reflected the findings of the laboratory studies. Approximately two decades ago, a study with silymarin supplementation (600 mg/day for 12 months) was conducted in insulin-treated diabetics with alcoholic cirrhosis, and the study had encouraging results. Beyond the antioxidant effects, there was a reduction of insulin resistance, a decrease in endogenous insulin hypersecretion and a reduced need for exogenous insulin administration [152]. Corroborating this observation, 10 years later, 25 diabetic (but non-cirrhotic) patients treated with silymarin (600 mg/day, for 16 weeks) showed reductions in glycemia, glycated hemoglobin, and an improved lipid profile in liver biomarkers [153]. In addition, Hussain et al. [154] showed that silymarin (200 mg/day, for 12 weeks) could be an important adjuvant for improving the glycemic control target by increasing insulin sensitivity in peripheral tissues through sulfonylureas (glibenclamide). This finding was recently corroborated by a study in which type 2 DM patients aged 25–50 years old who were on stable medications were supplemented with silymarin (420 mg/day, for six weeks). These patients also showed improvements in some antioxidant indices (SOD, GPx and total antioxidant capacity) as well as decreased lipid peroxidation besides hs-CRP levels without reporting any adverse effects of silymarin treatment [155]. Based on these results, more studies are still needed for the evaluation of the possible synergistic effects of silymarin with other antidiabetic classes (e.g., biguanides/metformin, dipeptidyl peptidase-4 inhibitors/sitagliptin; glucagon-like peptide-1 analogues/liraglutide; sodium-glucose cotransporter 2 inhibitors/dapagliflozin). Interestingly, silymarin has also been demonstrated to be an alternative treatment for diabetic renal patients who are using the maximum doses of angiotensin-converting enzyme (ACE) inhibitors or AT1 antagonists; even after a short duration treatment with silymarin (420 mg/day, for 12 weeks), these patients showed a reduction of 50% in albuminuria, urinary TNFα levels besides serum and urinary lipid peroxidation [156,157], which reflects a potential nephroprotective activity.

Although clinical trials with these polyphenols are still insufficient to define the optimal doses for treatment, the dosing range of silymarin used for diabetic patients is the closest to the ideal (when compared to diverse doses of resveratrol and quercetin) because it has clinically been investigated in several studies since the 1970s [143,158] compared to quercetin in 1995 [159] and resveratrol in 2007 [160]. The literature still describes that the therapeutic dose for the benefits of silymarin ranges between 210–800 mg/day, and silymarin appears to be safe and well tolerated up to 2100 mg/day [161], which reflects a wide therapeutic index [162]. Another additional advantage offered by silymarin in DM compared to other isolated polyphenols might be related to the relevance of silymarin use in combination with other antioxidants, which prevents individual antioxidant vulnerability and promotes synergistic effects against the chronic oxidative stress induced by diabetes [15].

## 4. Beneficial Effects of Probiotics: Highlights of Treatments with Kefir

In recent years, besides traditional drug treatments for DM, many efforts have been made in complementary or adjuvant therapy for the treatment of this complex disease [163]. Inadequate human dietary changes have been thought to be of major importance for the increased prevalence of DM. Overall, DM is estimated to afflict 350 million people globally and cost hundreds of billions of dollars annually [1,2]. Millions of cases could be prevented by including dietary modification to functional nutrition, which is a primary option for preventing metabolic disturbances and for reducing undesirable outcomes in DM [164,165]. Intestinal microbiota is a relevant therapeutic source for treatment of different diseases. Although there have been proposed different strategies including pre/probiotics and fecal microbiota transplantation interventions [166], in this section we review the main experimental and clinical studies that have focused on the beneficial effects of dairy cultured probiotics (live microorganisms) as coadjuvants in the prevention/treatment of this metabolic disorder.

Although many studies have been focused on the identification and use of innately occurring dairy components for the prevention and correction of metabolic dysfunctions accompanying DM [167], there is a growing and remarkable body of research showing the beneficial effects of non-innately cultured probiotics or bioactive end products [168]. These health benefits are achieved by stimulating beneficial gastrointestinal indigenous microflora proliferation [169]. Fermented milk kefir, which originated in the Northern Caucasus Mountains, is now commercially available in some countries, and in others it has been domestically produced and is spreading hand-to-hand [170]. The probiotic kefir has been associated with a range of health benefits, which have been reviewed by others [171], and its continuous intake has been shown to modulate complex cardiovascular and metabolic dysfunctions, including arterial hypertension [172] and DM [164].

In contrast with non-cultured dairy products, kefir grains are small clusters of microorganisms held together by an exopolysaccharide matrix named kefiran, which is the main functional component of the beverage [169,173,174,175]. Kefir grains are produced during the fermentation of milk by a complex symbiotic mixture of yeasts as well as lactic and acetic acid bacteria [170,173,174,176]. The dominating populations of bacterial genera in cultured kefir are *Lactobacillus*, *Lactococcus* and *Streptococcus* [169].

Rats administered with STZ (type 1 DM) or fed a hypercaloric diet (type 2 DM) are experimental models of DM [177]. In STZ-induced DM, it has been shown that daily administration of kefir caused an improvement in the increased levels of glycemia and glucose tolerance compared to conventional fermented milk [168,178,179]. Interestingly, kefiran, which is an exopolysaccharide isolated from kefir grains, has been shown to decrease blood pressure and blood glucose in animal models of hypertension [180] and an animal model of intolerance to glucose overload [181]. Kefiran-kefir also enhanced glucose uptake into insulin-responsive muscle cells, probably through activation of PI 3-kinases or another related signaling pathway [182]. Kefir also decreased polyuria, polydipsia and polyphagia [178]. In this model of DM, it has also been shown that administration of kefir results in a decrease in total cholesterol, triglycerides, LDL-cholesterol and an increase of HDL-cholesterol levels [179]. Moreover, kefir treatment of type 1 DM rats led to a decrease in the pro-inflammatory cytokines IL-1 and IL-6 as well as an increase of anti-inflammatory IL-10 compared to control groups [167]. These studies support the concept that kefir can be useful as a complementary or adjuvant therapy for better control of glycemia. However, the mechanisms by which probiotic kefir modulate hyperglycemia are not fully understood.

The beneficial effects of cultured probiotics have also been demonstrated in experimental type 2 DM. Administration of a strain of the probiotic microorganism *Lactococcus lactis* in rats with type 2 DM induced by a high-fructose diet resulted in significantly lower fasting blood glucose, HbA1c, insulin, free fatty acids and triglyceride levels than untreated DM rats [183]. By reducing the hyperglycemia, insulin resistance and hyperlipidemia, kefir actions were reflected in amelioration of the intracellular metabolic imbalance. In untreated DM animals, the excessive production of ROS overwhelmed the endogenous antioxidant defenses and resulted in oxidative stress, but this sequence of events can be attenuated through kefir treatment [183].

The use of antioxidant agents for therapeutic approaches in DM has been an attractive focus [184,185]. Accordingly, Friques et al. [172] observed through flow cytometry assays that kefir attenuated the endothelial dysfunction of spontaneously hypertensive rats by reducing the production of ·$O_{2}^{-}$, ONOO^−^ and H_2_O_2_. Augmented oxidative stress has also been shown to play a role in DM [185,186,187,188] and arterial hypertension [172]. First, it was shown that kefir reduced the intracellular levels of ROS in insulin-responsive muscle cells [182]. Second, the antioxidative effects of kefir in STZ-induced DM led to an improvement in the ROS levels [178,179]. The antioxidative effect seems to be the main mechanism by which kefir reduced proteinuria and azotemia, which consequently improved the progression of renal injury in type 1 DM rats [178]. These results indicated that kefir treatment may exert beneficial effects on the oxidative stress that accompanies DM and suggests it could be used as a non-pharmacological adjuvant to delay the progression of this disease [178].

Recently, our laboratory has assessed the actions of kefir on cardiac dysautonomia and impaired baroreflex control of cardiovascular function in SHR [189]. The main action of kefir on cardiac autonomic imbalance and impaired baroreflex appears to be through attenuation of the cardiac and vascular sympathetic hyperactivity as well as augmenting cardiac parasympathetic hypoactivity [189]. Some of these effects are also expected to occur in animal models of diabetes because they present similar disturbances in the cardiovascular system [190]. For example, in the model of type 1 DM, an important imbalance of the cardiac autonomic nerves, located at both tissues and molecular pathways, has been observed. Recently, in the first 10 weeks of experimental DM, a marked cardiac dysfunction and an incomplete recovery of the cardioinhibitory vagal nerves, accompanied by a remodeling process in the stimulatory noradrenergic nerves [191], have been shown.

Most clinical studies, including trials, have been conducted with patients who have type 2 DM, and most were treated with probiotic fermented milk kefir containing one, two or multi-strains of bacteria, such as *Lactobacillus casei*, *L. acidophilus*, *L. bulgaricus*, *Streptococcus thermophiles* and *Bifidobacterium lactis* [169]. The kefir effects observed on primary outcomes included decreased fasting blood glucose and HbA1c levels as well as improved insulin resistance [166,192,193,194]. The latter effect could be a consequence of a kefir-induced reduction in the inflammatory response [192]. In agreement with these results, it has been shown that kefir reduced pro-inflammatory cytokines, including TNFα, in DM [168,195]. The secondary outcomes included an improved lipid profile, blood pressure and hs-CRP, but kefir administration did not significantly change these parameters [191]. In contrast with experimental studies, it is still not clear whether kefir has beneficial effects on the lipid profile. An important finding after comparing the quantity of *Lactobacillus* and *Bifidobacterium* before and after the intervention was that there was successful passage of the probiotic supplement through the gastrointestinal tract [166,196]. The above studies support the concept that kefir can be useful as a complementary or adjuvant therapy for a better control of glycemia, insulin resistance and kidney function in diabetic individuals.

An important characteristic of DM is endothelial dysfunction, which has been shown in experimental and clinical studies [197]. Our laboratory has demonstrated that kefir administered for at least 60 days in spontaneously hypertensive rats resulted in a significant attenuation of endothelial dysfunction [172]. Therefore, there is a need for more studies to test the hypothesis that kefir administration also exhibits benefits against this abnormality.

A limitation in the therapeutic use of the probiotic kefir in DM is that this is a heterogeneous and a multiple systems-derived disease that results in multiple complications. Therefore, it makes it hard to prevent or to treat DM with traditional medicine and functional nutrition, especially when treating different people with different needs. However, there is evidence that kefir has great potential to become an adjuvant alternative for control of glycemia and other diabetes-related outcomes. Further studies are needed not only to clarify the mechanisms behind the effects of kefir but also to determine which microorganisms in kefir are responsible for its benefits.

## 5. Beneficial Effects of Phosphodiesterase Inhibitors in Diabetes Mellitus: New Insights

Several investigations have demonstrated that increased cyclic GMP (cGMP) signaling might be an important strategy for reducing the progression of diabetes through multiple pathways [198]. Concerning glycemic control, even if an increase in intracellular calcium is the principal signal that activates insulin exocytosis, cGMP may also participate through distinct signals [199,200] and potentiating the stimulation of glucose [201,202,203]. In parallel, some in vitro studies have shown that cGMP may enhance insulin sensitivity in target organs (muscle and adipocytes) by stimulating GLUT4 recruitment into the plasma membrane [204,205,206]. In addition, because NO/cGMP signaling is fundamental to vascular protection [207,208,209], the increment of this pathway may be an attractive strategy to attenuate endothelial dysfunction development in diabetic complications, which is as major cause of disability and death in patients with DM [4,44].

**Figure 1:**
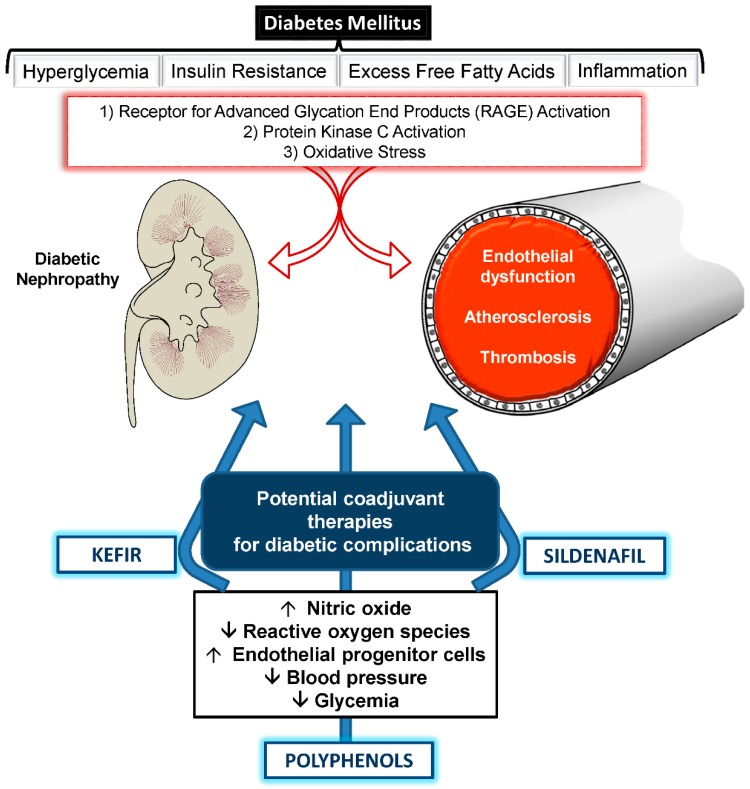
Schematic representation of metabolic complications of diabetes mellitus in two important target organs, and the main effects exhibited by three important coadjuvants currently under investigation with the aim of preventing and treating this complex disease. Arrows up (↑): increase; arrows down (↓): decrease.

Pharmacological strategies to increase cGMP signaling may be achieved through two main routes: (1) direct activation of guanylate cyclase directly by augmentation of NO; and/or (2) decreasing cGMP hydrolysis through PDE5 inhibitors (sildenafil/Viagra™, vardenafil/Levitra™, tadalafil/Cialis™, avanafil/Stendra™), which is currently considered an important tool to treat endothelial dysfunction in DM [209,210,211]. Because PDE5 is expressed in some tissues of the body (e.g., corpus cavernosum, platelets, systemic arteries and veins) [209,212], the diminished NO bioavailability in diabetic vasculature can be partially compensated through PDE5 inhibitors. Interestingly, although some recent studies demonstrated that association with antioxidants (e.g., polyphenols or vitamin E) potentiates vascular protection [213,214], sildenafil may also provide intrinsic antioxidant effects through NADPH oxidase activity inhibition [215]. This evidence was complemented by our research in various models of hypertension, nephropathy or atherosclerosis and demonstrated protective effects for endothelial, cardiac and kidney functions in physiological parameters, such as morphological analyses (Figure 1) [209,212,216,217,218,219]. Therefore, our studies and other experimental evidence support the clinical findings of improvement in endothelial function, reduction of markers of vascular inflammation, and beneficial effects for conditions beyond erectile dysfunction [220,221,222,223,224].

In relation to glycemic control, PDE5 inhibitors may have improved insulin sensitivity both in isolated human endothelial cells [225] and in high fat-fed mice [226], which corroborates the results related to cGMP that were previously discussed. Moreover, recent findings showed that chronic treatment with tadalafil reduced inflammatory cytokines, infarct size and oxidative stress in the hearts of diabetic mice by reducing NADPH oxidase activity, oxidized glutathione and lipid peroxidation [227,228]. These results show a potential role for PDE5 inhibitors in treating diabetes-related cardiac and inflammatory complications. Interestingly, clinical investigations confirmed that sildenafil could improve insulin sensitivity in addition to fibrinolytic balance and albuminuria in hyperglycemic patients [198,229]. These studies suggest that PDE inhibitors can be effective (and safely used) in patients with multiple comorbidities and therapies, except for patients treated with continuous nitrates [209,223,230].

## 6. Conclusions

In the present review, besides showing the importance of lifestyle modification, diet and weight control to prevent DM and its aggravation, we highlight recent experimental and clinical evidences of potential coadjuvants in the management of DM without compromising the function of β-cells via hyperinsulinism. Here, we have discussed the substances that exhibit direct/indirect or pleiotropic effects on glycemic control in DM and on oxidative stress that is one of the most contributors to the complications of this disease by affecting the different target organs. Among several options, we have highlighted new promising therapeutic coadjuvants, including the cultured probiotic microorganisms (such as kefir grains) and the PDE5 inhibitors (such as sildenafil), which besides the reduction of hyperglycemia and ameliorate insulin resistance, they have been shown to reduce the oxidative stress and improved the endothelial dysfunction in the systemic vascular circulation. In the near future, there are expected experimental studies designed to clear the intracellular pathways involving those coadjuvants discussed in this review as well as promoting clinical trials that may contribute to new strategies against the harmful effects of diabetic complications.

## Abbreviations

ACE	angiotensin-converting enzyme
AGEs	glycation end products
cGMP	cyclic guanosine monophosphate
COX	cyclooxygenase
CRP	C-reactive protein
CVD	cardiovascular diseases
CYP450	cytochrome P450
DM	diabetes mellitus
ECM	extracellular matrix
eGFR	estimated glomerular filtration rate
eNOS	endothelial nitric oxide synthase
GLP-1	glucagon-like peptide-1
GLUT	glucose transporter
GPx	glutathione peroxidase
H_2_O_2_	hydrogen peroxide
HbA1c	glycated hemoglobin level
HDL	high density lipoprotein
hs-CRP	high-sensitivity C-reactive protein
IGT	impaired glucose tolerance
IL	interleukin
iPDE	PDE inhibitors
LDL	low density lipoprotein
NADH	nicotinamide adenine dinucleotide
NADPH	nicotinamide adenine dinucleotide phosphate
NF-κB	nuclear factor-κB
NHAHES	national health and nutrition examination survey
NO	nitric oxide
NOS	nitric oxide synthase
Nox	NADPH oxidases
·$O_{2}^{-}$	superoxide anion
ONOO^−^	peroxynitrite
OH^−^	hydroxyl
·OH	hydroxyl radical
PARP	poly ADP ribose polymerase
PDE5	phosphodiesterase 5
PKC	protein kinase C
PPARγ	proliferator-activated receptor γ
ROS	reactive oxygen species
SHR	spontaneously hypertensive rats
SIRT	sirtuin 1
SOD	superoxide dismutase
STZ	streptozotocin
TNFα	tumor necrosis factor
